# Supplementary material for: Impact of fitness coach behavior on exercise motivation, commitment, and enjoyment: A longitudinal study
Source: PLoS One. 2024 Dec 30;19(12):e0310931. doi: 10.1371/journal.pone.0310931 (PMC11684697; doi:10.1371/journal.pone.0310931)
Supplement: Attachment — Submitted filename: Editor_letter.docx [file pone.0310931.s001.docx]

Dear Editor,

We hope that this letter finds you well. Thank you for the attention given to our manuscript. In the course of your comments in the email, efforts were taken to address the raised issues, in particular:

1. Amendments have been made to address additional requirements, namely relative to the requirements for the manuscript style requirements.
2. The Data Availability Statement should include the explicit description: “All relevant data are within the manuscript”. An oversight was made in include a reference to “supporting information files”. Indeed, we confirm that the entirety of the information is encompassed in the manuscript. However, if there are any remaining doubts and people desire additional information, as is common procedure we are entirely available to address any inquiries via email contact.

Thank you again for your attention and contribution to our manuscript. Hope to hear from you soon.
